# Impact of Simultaneous Consideration of Cardiac and Vascular Function on Long-Term All-Cause and Cardiovascular Mortality

**DOI:** 10.3390/jcm8122145

**Published:** 2019-12-04

**Authors:** Po-Chao Hsu, Wen-Hsien Lee, Wei-Chung Tsai, Chun-Yuan Chu, Ying-Chih Chen, Meng-Kuang Lee, Tsung-Hsien Lin, Chee-Siong Lee, Wen-Chol Voon, Wen-Ter Lai, Sheng-Hsiung Sheu, Ho-Ming Su

**Affiliations:** 1 Division of Cardiology, Department of Internal Medicine, Kaohsiung Medical University Hospital, Kaohsiung 80756, Taiwan; 2 Faculty of Medicine, College of Medicine, Kaohsiung Medical University, Kaohsiung 80708, Taiwan; 3 Department of Internal Medicine, Kaohsiung Municipal Siaogang Hospital, Kaohsiung 812, Taiwan

**Keywords:** all-cause mortality, arterial stiffness, cardiovascular mortality, left ventricular systolic function, pulse wave velocity

## Abstract

Background: Left ventricular ejection fraction (LVEF) is a good indicator of cardiac function, and brachial-ankle pulse wave velocity (baPWV) is a good indicator of vascular function. Both of them can predict cardiovascular (CV) outcomes. Objectives: There is scarce literature discussing the impact of simultaneous consideration of cardiac and vascular function on overall and CV mortality. Methods: We included 958 patients and classified them into four groups. Groups 1 to 4 were patients with LVEF ≥ 50% and baPWV below the median, LVEF < 50% but baPWV below the median, LVEF ≥ 50% but baPWV above the median, and LVEF < 50% and baPWV above the median, respectively. Results: The median follow-up to mortality was 93 (25th–75th percentile: 69–101) months. There were 91 cases of CV mortality and 238 cases of all-cause mortality. After multivariable analysis, age, gender, diabetes, mean blood pressure, group 2 versus group 1, and group 4 versus group 1 were significant predictors of all-cause mortality (P ≤ 0.038) and age, diabetes, mean blood pressure, group 2 versus group 1, and group 4 versus group 1 were significant predictors of CV mortality (P ≤ 0.008). Conclusions: Patients with higher LVEF and lower baPWV had a similar overall and CV mortality as patients with higher LVEF and baPWV. Patients with lower LVEF and higher baPWV had the highest overall and CV mortality among the four study groups. In addition, patients with lower LVEF alone had a higher CV mortality than the patients with higher baPWV alone. Therefore, simultaneous consideration of cardiac and vascular function may be useful in predicting overall and CV mortality.

## 1. Introduction

Left ventricular systolic function is a good indicator of cardiac function. Left ventricular ejection fraction (LVEF) is a widely used parameter for the assessment of cardiac systolic function and has been established as a powerful predictor of adverse cardiovascular (CV) outcomes [1,2,3,4,5,6,7,8,9,10,11]. 

Increased brachial-ankle pulse wave velocity (baPWV), which reflects increased arterial stiffness, is associated with the presence of coronary artery narrowing [12] and is also regarded as an independent predictor of CV outcomes [13,14,15,16]. Arterial stiffness occurs as a consequence of biological aging and arteriosclerosis. It is considered to be the loss of arterial elasticity, which represents the mechanical property of artery resistant to deformation [17]. Therefore, arterial stiffness can be regarded as vascular dysfunction due to arterial stiffening. 

CV dysfunction progresses while there are interactions between vascular and cardiac system, but this progression has different speeds in vessels and the heart. For example, in some cases, dysfunction of vessels progresses first, and in other cases, dysfunction of the heart progresses first. In either case, patients can finally develop CV dysfunction as a result [18]. However, there is scarce literature discussing the impact of simultaneous consideration of cardiac and vascular function on the long-term all-cause and CV mortality. Therefore, the aim of this study was to evaluate the long term mortality among patients with different cardiac or vascular function. 

## 2. Materials and Methods

### 2.1. Study Population

Study population was enrolled from a group of patients arranged for examinations of echocardiography at Kaohsiung Municipal Siaogang Hospital from March 2010 to March 2012 because of suspected hypertension, coronary artery disease, heart failure, abnormal cardiac physical examinations, a survey for dyspnea, or a pre-operative cardiac function survey. These patients were referred from the inpatient and outpatient service if they fulfilled the above criteria. We excluded the subjects with significant atrial fibrillation, diseases of mitral and aortic valve, or inadequate image visualization. In order to minimize the influence of blood pressure and heart rate on the results of echocardiographic and baPWV studies caused by different measurement time, we shortened the time between echocardiographic and baPWV measurements. Hence, we did not include patients consecutively because baPWV has to be measured within 10 minutes after the completion of an echocardiographic examination. We did not include patients if their baPWV measurement could be performed within 10 min after the completion of echocardiographic examination. Finally, 958 patients were enrolled. The study population was further divided into four groups using LVEF and baPWV measurements. Groups 1, 2, 3, and 4 were made up of patients with LVEF ≥ 50% and baPWV below the median, LVEF < 50% but baPWV below the median, LVEF ≥ 50% but baPWV above the median, and LVEF < 50% and baPWV above the median, respectively. 

### 2.2. Ethics Statement

Our study protocol was approved by the institutional review board committee of the Kaohsiung Medical University Hospital (KMUH-IRB). Informed consent was obtained from the patients and our study was conducted according to the principles expressed in the Declaration of Helsinki. 

### 2.3. Echocardiographic Evaluation

The examinations of echocardiography were performed by one experienced physician using the machine of echocardiography (Vivid 7, General Electric Medical Systems, Horten, Norway), and the patients lay down in the left decubitus position. We measured LVEF by using the modified Simpson’s method.

### 2.4. Measurement of baPWV

The baPWV was measured by using a device (VP1000, Colin, Aichi, Japan) which simultaneously measures blood pressure in both arms and ankles. [19,20] The baPWV was calculated as the transmitted distance from the pulse wave from brachial to tibial arteries divided by passage time of the pule wave. After getting the bilateral baPWV, the two values were averaged for further analysis.

### 2.5. Collection of Medical and Demographic Data

Medical and demographic data including age, gender, and other comorbid diseases were collected from medical records or interviews with patients. For comorbid diseases, diabetes was defined as fasting blood sugar ≥ 126 mg/dL or anti-diabetic agent usage. Cardiovascular disease (CVD) was defined as having the history of coronary artery disease, cerebrovascular disease, or peripheral artery disease. Mean blood pressure was calculated as 2/3 diastolic blood pressure plus 1/3 systolic blood pressure. Details on the use of medications including antiplatelet, β-blockers, calcium channel blockers, angiotensin converting enzyme inhibitors, angiotensin II receptor blockers, and diuretics at the time of patient enrollment were obtained from medical records.

### 2.6. Definition of all-cause and CV mortality

All study participants were followed up until December 2018. Survival information and causes of death were obtained from the official death certificate and final confirmation by the Ministry of Health and Welfare. The causes of death were classified by the International Classification of Diseases 10th Revision. Causes of CV mortality were defined deaths due to hypertensive disease, cardiac disease, cerebral vascular disease, ischemic heart disease, myocardial infarction, heart failure, valvular heart disease, and atherosclerotic vascular disease (Supplement Table S1). No participant was lost during the follow-up in our study.

### 2.7. Statistical Analysis

We used SPSS 22.0 software for statistical analysis. Power and sample size were calculated by PASS software and at least 823 subjects were needed in our study if power was 0.9. Our data was expressed as mean ± standard deviation, percentage, or median (25th–75th percentile) for follow-up period. Multiple comparisons among study groups were done by one-way analysis of variance followed by a post hoc test. Categorical and continuous variables between groups were compared by Chi-square test and independent samples t test, respectively. Age, gender, mean blood pressure, and the significant variables in the univariable analysis were selected for multivariable analysis. Time to the CV and overall mortality events and covariates of risk factors were modeled using the Cox proportional hazards model with forward selection. The incremental value of LVEF and baPWV over conventional parameters to predict overall and CV mortality was studied by calculating the improvement in global Chi-square value. If the incremental value of Chi-square value ≥ 4, then the P value should be significant (*P* < 0.05). Survival plot was calculated from baseline to time of mortality events. All tests were 2-sided and the level of significance was established as *P* < 0.05. 

## 3. Results

Among the 958 subjects, mean age was 62 ± 14 years. The median baPWV value was 1668 (25th–75th percentile: 1459–1992) cm/s. Mortality data were obtained from the Collaboration Center of Health Information Application (CCHIA), Ministry of Health and Welfare, Executive Yuan, Taiwan. The follow-up period to mortality events was 93 (25th–75th percentile: 69–101) months in all patients. Mortality events were documented during the follow-up period, including CV mortality (*n* = 91) and overall mortality (*n* = 238).

We showed the comparison of clinical characteristics among the study groups in Table 1. There were 495, 62, 350, and 51 patients in groups 1 to 4, respectively. Significant differences among the four groups were found in the age, gender, prevalence of diabetes mellitus, mean blood pressure, body mass index, heart rate, LVEF, baPWV, and usage of aspirin, β-blockers, calcium channel blockers, angiotensin-converting enzyme inhibitors, and diuretics. The study was conducted with patients taking their usual medications.

Table 2 shows the predictors of all-cause mortality using the Cox proportional hazards model. In the univariable analysis, age, diabetes mellitus, total cholesterol, heart rate, diuretic use, and study group difference (group 2 versus group 1, group 3 versus group 1, group 4 versus group 1, all *P* < 0.001) are significant predictors of all-cause mortality. Furthermore, in the multivariable analysis, only age (*P* < 0.001), gender (*P* = 0.007), diabetes mellitus (*P* < 0.001), mean blood pressure (*P* = 0.003), and study group difference (group 2 vs. group 1, *P* = 0.038; group 4 vs. group 1, *P* < 0.001) could predict all-cause mortality. 

Table 3 shows the predictors of CV mortality using the Cox proportional hazards model. In the univariable analysis, age, diabetes mellitus, heart rate, diuretic use, and study group difference (group 2 versus group 1, group 3 versus group 1, group 4 versus group 1, all *P* < 0.001) were significant predictors of CV mortality. Furthermore, in the multivariable analysis, only age (*P* < 0.001), diabetes mellitus (*P* < 0.001), mean blood pressure (*P* = 0.008), and study group difference (group 2 versus group 1; group 4 versus group 1, both *P* < 0.001) could predict CV mortality. 

In addition, we used LVEF and baPWV instead of the study groups to evaluate the long term all-cause and CV mortality prediction. The result is shown in Table 4. We used LVEF or baPWV for the initial model. After different multivariable adjustments, LVEF and baPWV were still independent predictors of all-cause and CV mortality.

Other confounders for all-cause mortality prediction were age, gender, diabetes mellitus, total cholesterol, mean blood pressure, heart rate, and diuretic use. Other confounders for cardiovascular mortality prediction were age, gender, diabetes mellitus, mean blood pressure, heart rate, and diuretic use.

We also used other different groups as reference groups in our multivariable Cox proportional hazards model to predict all-cause and CV mortality. In the analysis, group 4 had the highest CV and all-cause mortality among 4 groups. Group 2 had a higher all-cause mortality than group 1 and a higher CV mortality than groups 1 and 3. 

**Figure 1:**
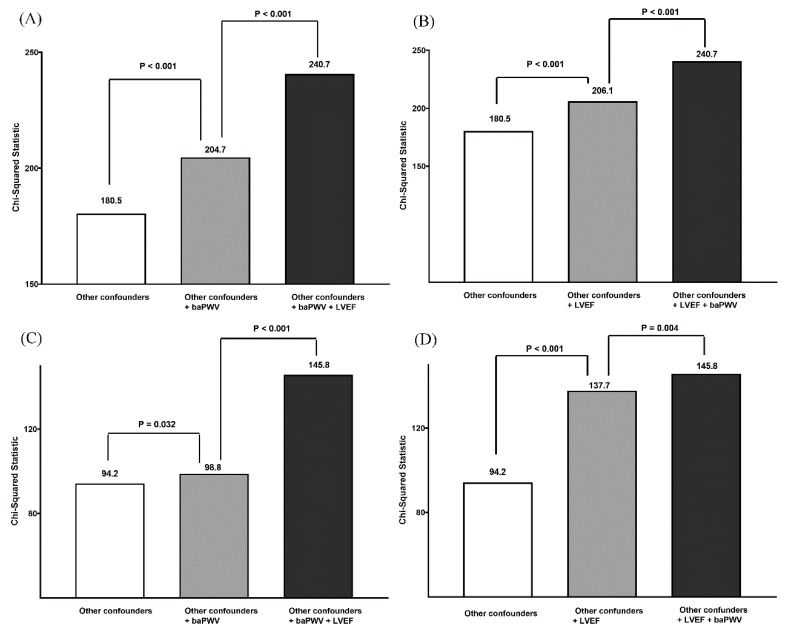
The additive effect of using both left ventricular ejection fraction (LVEF) and brachial-ankle pulse wave velocity (baPWV) measures on overall mortality (**A**,**B**) and cardiovascular mortality (**C**,**D**) prediction by calculating the improvement in global Chi-square value. Other confounders in Figure 1A,B included age, gender, diabetes mellitus, total cholesterol, mean blood pressure, heart rate, and diuretic use. Other confounders in Figure 1C,D included age, gender, diabetes mellitus, mean blood pressure, heart rate, and diuretic use. All of the incremental values of Chi-square value were > 4 in Figure 1A–D, so the *P* values were all significant (*p* ≤ 0.032).

**Figure 2:**
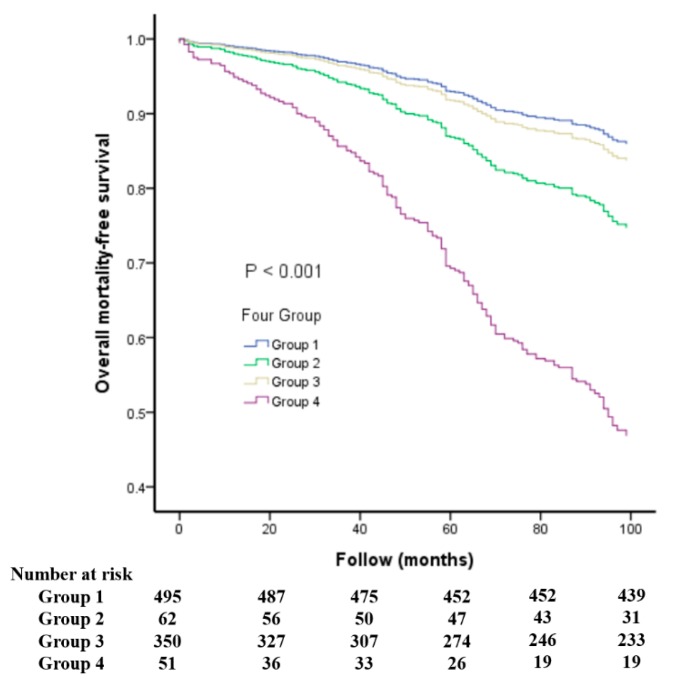
The survival curves for overall mortality-free survival among four groups. Group 1: LVEF > 50%, baPWV below the median; Group 2: LVEF < 50%, baPWV below the median; Group 3: LVEF > 50%, baPWV above the median; Group 4: LVEF < 50%, baPWV above the median.

**Figure 3:**
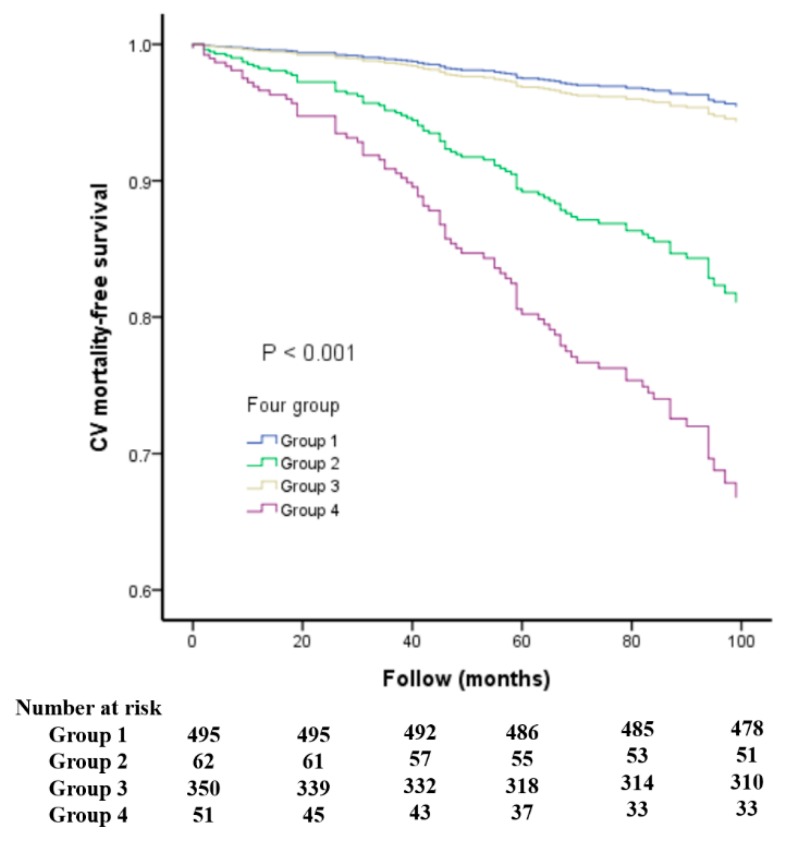
The survival curves for cardiovascular (CV) mortality-free survival among four groups. Group 1: LVEF > 50%, baPWV below the median; Group 2: LVEF < 50%, baPWV below the median; Group 3: LVEF > 50%, baPWV above the median; Group 4: LVEF < 50%, baPWV above the median.

Figure 1 shows the additive effect of using both baPWV and LVEF measures on overall mortality (Figure 1A,B) and CV mortality (Figure 1C,D) prediction by calculating the improvement in global Chi-square value. The variables in the other confounders of Figure 1A,B included age, gender, diabetes mellitus, total cholesterol, mean blood pressure, heart rate, and diuretic use. After gradually adding baPWV or LVEF into other confounders, we found that other confounders + baPWV or LVEF had a better predictive value for all-cause mortality than other confounders and other confounders + LVEF + baPWV had a better predictive value for all-cause mortality than other confounders + baPWV or LVEF. The variables in the other confounders of Figure 1C,D included age, gender, diabetes mellitus, mean blood pressure, heart rate, and diuretic use. After gradually adding baPWV or LVEF into other confounders, we found that other confounders + baPWV or LVEF had a better predictive valve for CV mortality than other confounders and other confounders + LVEF + baPWV had a better predictive value for CV mortality than other confounders + baPWV or LVEF. In addition, Figure 2 and Figure 3 illustrate the survival curves for overall and CV mortality-free survival among the four groups, respectively.

## 4. Discussion

The main objective of our study was to evaluate the long term all-cause and CV mortality among four groups classified by LVEF and baPWV values, especially between groups 2 and 3. Our results showed that group 4 had the highest all-cause and CV mortality among the four study groups. Group 1 had a similar overall and CV mortality to that of group 3. In addition, group 2 had a higher CV mortality than group 3, but this result was not found in all-cause mortality.

As we previous mentioned, low LVEF is a good indicator of cardiac dysfunction which is widely used to predict future CV outcomes [1,2,3,4,5,6,7,8,9,10,11]. The mortality rate after diagnosis of heart failure was about 10% at 30 days, 20–30% at 1 year, and 45–60% over 5 years based on the Framingham Heart Study. Lee et al. reported that CV death occurred in 44.5% and 69.9% patients with heart failure with preserved and reduced LVEF, respectively [21]. Many studies of heart failure showed that patients with reduced LVEF had a higher mortality than patients with preserved LVEF [22,23]. In our study, we used LVEF 50% as the cut-off value because current guidelines regarded LVEF 40–50% as the mid-range LVEF and several research examples also used LVEF 50% as the cut-off value [24,25]. In addition, high baPWV is considered as a good indicator of vascular dysfunction and is also an independent predictor of CV morbidity and mortality in the elderly and general population, patients with hypertension, and patients with end-stage renal disease [14,15,16]. In our study, we used baPWV as the parameter of arterial stiffness. Tanaka et al. reported that baPWV and carotid-femoral pulse wave velocity were indices of arterial stiffness showing similar extents of associations to CV disease risk factors and clinical events [26].

Before evaluation of long term survival, clarifying the characteristics of the four groups is very important. Patients in group 1 had a relatively normal CV function. Group 1 was relatively younger and had a lower percentage of CVD (20.8%). Patients in group 2 had cardiac dysfunction alone. Group 2 members were also younger but had a higher percentage of CVD (54.8%). Patients in group 3 had vascular dysfunction. Group 3 members were older and had the highest percentage of hypertension but a lower percentage of CVD (26.0%). Although patients in group 1 had the smallest percentage of CVD, there was no significant difference between group 1 and group 3. Patients in group 4 had CV dysfunction. Group 4 members were also older and had a higher percentage of CVD (51.0%). Cardiac and vascular dysfunction were reported to be independent prognostic predictors (Supplement Table S2), but there was scarce literature discussing the impact of combined consideration of cardiac and vascular dysfunction on the long term all-cause and CV mortality. In fact, our present study found that patients with CV dysfunction had the highest all-cause and CV mortality. Through additional consideration of baPWV, we could further identify the highest risk group in patients with reduced LVEF. Patients with cardiac dysfunction alone had a higher CV mortality than patients with vascular dysfunction alone. This finding was reasonable because the patients with reduced LVEF could have a higher CV mortality risk than the patients with preserved LVEF. In addition, by comparison of Chi-square values, we found that combined consideration of LVEF and baPWV had a more extra benefit in prediction of all-cause and CV mortality than consideration of LVEF or baPWV alone. Hence, simultaneous consideration of cardiac and vascular function was actually helpful in the improvement of survival prediction.

### Study Limitations

First, the majority of our patients were treated with CV drugs. For ethical reasons, we did not withdraw these medications. Hence, we could not exclude the influence of CV drugs on our study. However, we already adjusted the associated usage of CV drugs in the multivariable analysis. Second, our study was aimed to evaluate the mortality events, so non-fatal events were not studied. Third, because there was no study to document a reliable value of baPWV in indicating vascular dysfunction, a median value of baPWV was used to classify our study patients. Fourth, because many patients in our study did not have B-type Natriuretic Peptide (BNP) or NT-proBNP data, it was difficult to diagnose the cases of heart failure with preserved LVEF. Therefore, we did not further discuss the issue about heart failure with preserved LVEF.

## 5. Conclusions

In our study, patients with lower LVEF and higher baPWV had the highest all-cause and higher CV mortality than other patient groups. In addition, patients with lower LVEF alone had a higher CV mortality than the patients with higher baPWV alone. Therefore, combined consideration of cardiac and vascular function may be useful in prediction of long term all-cause and CV mortality.

## Figures and Tables

**Table 1:** Comparison of clinical characteristics among study groups.

	Group1 (LVEF ≥ 50%, baPWV below the Median)	Group2 (LVEF < 50%, baPWV below the Median)	Group 3 (LVEF ≥ 50%, baPWV above the Median)	Group 4 (LVEF < 50%, baPWV above the Median)	*p* Value (ANOVA among Groups)	All Patients
Number	495	62	350	51		958
Age (years)	56 ± 13	58 ± 16	70 ± 11 *^,$^	71 ± 12 *^,$^	<0.001	62 ± 14
Male Gender (%)	59.0	75.4 *	47.6 *^,$^	60.8	<0.001	55.8
DM (%)	21.6	35.5 *	37.7 *	31.4	<0.001	28.9
CVD (%)	20.8	54.8 *	26.0 ^$^	51.0 *^,#^	<0.001	26.5
Mean blood pressure	92 ± 12	87 ± 12 *	103 ± 13 *^,$^	100 ± 15 *^,$^	<0.001	96 ± 14
Total cholesterol	192 ± 39	176 ± 50	191 ± 40	183 ± 46	0.07	190 ± 41
BMI (kg/m^2^)	26.8 ± 4.0	26.5 ± 4.7	25.9 ± 3.8 *	24.4 ± 3.5 *^,$^	<0.001	26.3 ± 4.0
Heart rate (beats/min)	67 ± 10	75 ± 17 *	70 ± 12 *^,$^	79 ± 17 *^,#^	<0.001	69 ± 12
LVEF (%)	67 ± 8	38 ± 9 *	67 ± 8 ^$^	38 ± 8 *^,#^	<0.001	64 ± 12
baPWV (cm/s)	1475 ± 173	1456 ± 178	2163 ± 387 *^,$^	2278 ± 668 *^,$^	<0.001	1768 ± 466
Numbers of Mortality						
All-cause	58	23	120	37	<0.001	238
Cardiovascular	17	13	41	20	<0.001	91
Medications						
Aspirin (%)	30.2	47.5 *	33.9 ^$^	41.2	0.026	33.1
β-blockers (%)	40.7	67.2 *	38.1 ^$^	35.3 ^$^	<0.001	41.0
CCBs (%)	38.2	19.7 *	46.4 *^,$^	25.5 ^#^	<0.001	39.2
ACEIs (%)	5.9	21.3 *	9.5 ^$^	11.8	<0.001	8.5
ARBs (%)	47.3	50.8	50.7	45.1	0.72	48.5
Diuretics (%)	24.9	59.0 *	31.9 *^,$^	47.1 *^,#^	<0.001	30.7

ACEIs: angiotensin-converting enzyme inhibitors; ARBs: angiotensin II receptor antagonists; baPWV: brachial-ankle pulse wave velocity; BMI: body mass index; CCBs: calcium channel blockers; CVD: cardiovascular disease; DM: diabetes mellitus; LVEF: left ventricular ejection fraction. * *p* < 0.05 compared to group 1, ^$^ *p* < 0.05 compared to group 2, ^#^ *p* < 0.05 compared to group 3.

**Table 2:** Predictors of all-cause mortality using the Cox proportional hazards model.

Parameter	Univariate		Multivariate (Forward)	
	HR (95% CI)	*P*	HR (95% CI)	*P*
Age (+ 13.74 year)	2.949 (2.494–3.486)	<0.001	3.159 (2.524–3.952)	<0.001
Male gender (male vs. female)	1.109 (0.852–1.444)	0.44	1.573 (1.152–2.150)	0.004
Diabetes mellitus (yes vs. no)	2.275 (1.750–2.957)	<0.001	2.411 (1.773–3.278)	<0.001
Mean blood pressure (+13.80 mmHg)	1.120 (0.983–1.276)	0.09	1.300 (1.101–1.534)	0.002
Total cholesterol (−40.68 mg/dL)	0.741 (0.631–0.872)	<0.001	–	–
Heart rate (+12.20 beat/minute)	1.242 (1.096–1.407)	0.001	–	–
Smoking (ever vs. no)	0.775 (0.518–1.158)	0.21		
Medications				
Aspirin use	1.252 (0.954–1.642)	0.11		
Beta blocker use	1.037 (0.796–1.351)	0.79		
Calcium channel blocker use	1.127 (0.865–1.468)	0.38		
ACEI use	1.142 (0.736–1.773)	0.55		
ARB use	1.044 (0.804–1.354)	0.75		
Diuretic use	1.886 (1.449–2.454)	<0.001	–	–
Study Group		<0.001		<0.001
Group 2 vs. Group 1	3.810 (2.307–6.292)	<0.001	1.954 (1.049–3.638)	0.035
Group 3 vs. Group 1	3.459 (2.516–4.756)	<0.001	1.150 (0.764–1.730)	0.50
Group 4 vs. Group 1	10.854 (7.014–16.797)	<0.001	4.693 (2.745–8.022)	<0.001

The HRs of continuous variables were calculated as a standard deviation change. HR: hazard ratio; CI: confidence interval; other abbreviations as in Table 1.

**Table 3:** Predictors of cardiovascular mortality using Cox proportional hazards model.

Parameter	Univariate		Multivariate (Forward)	
	HR (95% CI)	*P*	HR (95% CI)	*P*
Age (+13.74 year)	3.035 (2.305–3.997)	<0.001	2.860 (2.103–3.889)	<0.001
Male gender (male vs female)	0.991 (0.647–1.516)	0.97	–	–
Diabetes mellitus (yes vs. no)	2.727 (1.789–4.157)	<0.001	2.746 (1.772–4.256)	<0.001
Mean blood pressure (+13.80 mmHg)	1.208 (0.982–1.487)	0.074	1.371 (1.086–1.731)	0.008
Total cholesterol (−40.68 mg/dL)	0.774 (0.592–1.012)	0.061		
Heart rate (+12.20 beat/minute)	1.309 (1.073–1.598)	0.008	–	–
Smoking (ever vs. no)	0.749 (0.388–1.448)	0.39		
Medications				
Aspirin use	1.318(0.852–2.039)	0.22		
Beta blocker use	1.271(0.834–1.938)	0.26		
Calcium channel blocker use	1.180(0.772–1.806)	0.45		
ACEI use ARB use	1.235(0.619–2.463) 1.283(0.841–1.956)	0.55 0.25	–	–
Diuretic use	1.909(1.248–2.922)	0.003	–	–
Study Group		<0.001		<0.001
Group 2 vs. Group 1	6.612 (3.096–14.120)	<0.001	4.518 (2.050–9.955)	<0.001
Group 3 vs. Group 1	3.881 (2.199–6.849)	<0.001	1.256 (0.661–2.387)	0.49
Group 4 vs. Group 1	20.443 (10.494–39.823)	<0.001	8.702 (4.283–17.679)	<0.001

The HRs of continuous variables were calculated as a standard deviation change. HR: hazard ratio; CI: confidence interval; other abbreviations as in Table 1.

**Table 4:** Predictors of all-cause and cardiovascular mortality using Cox proportional hazards model by adding baPWV and LVEF instead of a study group.

Model	Variable	All-Cause Mortality Prediction		CV Mortality Prediction	
		HR (95% CI)	*P*	HR (95% CI)	*P*
Model 1: LVEF	LVEF	0.648 (0.579–0.725)	<0.001	0.548 (0.464–0.648)	<0.001
Model 2: LVEF + other confounders	LVEF	0.655 (0.565–0.758)	<0.001	0.510 (0.423–0.614)	<0.001
Model 3: baPWV	baPWV	1.886 (1.713–2.076)	<0.001	1.831 (1.556–2.154)	<0.001
Model 4: baPWV + other confounders	baPWV	1.573 (1.356–1.825)	<0.001	1.272 (1.029–1.571)	0.026
Model 5: Other confounders + LVEF + baPWV	baPWV LVEF	1.640(1.416–1.899) 0.628(0.541–0.729)	<0.001 <0.001	1.462 (1.205–1.774) 0.507 (0.422–0.609)	<0.001 <0.001

The HRs of LVEF and baPWV were calculated as a standard deviation change. CV: cardiovascular; HR: hazard ratio; CI: confidence interval; other abbreviations as in Table 1.

## Supplementary Materials

The following are available online at https://www.mdpi.com/2077-0383/8/12/2145/s1, Table S1: Cause of cardiovascular mortality (ICD-10), Table S2: Summary of studies of evaluation of PWV in HF patients for outcome prediction.
