# Supplementary material for: Impact of Simultaneous Consideration of Cardiac and Vascular Function on Long-Term All-Cause and Cardiovascular Mortality
Source: J Clin Med. 2019 Dec 4;8(12):2145. doi: 10.3390/jcm8122145 (PMC6947191; doi:10.3390/jcm8122145)
Supplement: Supplementary file 1 [file jcm-08-02145-s001.pdf]

Supplement Table 1: Cause of cardiovascular mortality (ICD-10).

Cause of cardiovascular mortality	ICD-10
Hypertensive disease	I10-I15
Cardiac disease (Except hypertensive disease)	I01-I02.0, I05-I09, I20-I25, I27, I30-I52
Cerebrovascular disease	I60-I69
Atherosclerosis vascular disease	I70
Aortic aneurysm and dissection	I71

Supplement Table 2: Summary of studies of evaluation of PWV in HF patients for outcome prediction.

Study	Study population	Follow-up	Outcome	Ref
Sung SH et al. (2011)	80 patients with acute HF	174 ± 32 days	PWV were significantly lower in patients without events than those with events (HF rehospitalization /nonfatal MI/nonfatal stroke/ mortality).	1
Demir S et al. (2013)	98 HF patients with LVEF ≤ 35%	18 months	PWV was powerful determinants of mortality (OR: 0.853, p = 0.025)	2
Regnault V et al. (EPHESUS substudy) (2014)	306 patients with acute MI and HF with LVEF<40%,	16 months	Higher LVEF or pulse pressure associated with lower all-cause and CV mortality. Increased PWV was associated with higher all-cause and CV mortality	3
Tokitsu et al. (2018)	502 patients with HFpEF	Mean 1017 days	Patients with PWV > 1900 cm/s and < 1300 cm/s had a significant higher total CV events than 1300 ≤ PWV ≤ 1900 cm/s	4
Huang WM et al. (2019)	Cohort A: 230 patients (HFrEF, HFmrEF, HFpEF) Cohort B: 2677 patients	Mean 21.3 +/- 13.6 months	HFmrEF had higher PWV than reduced HFrEF HFmrEF and HFrEF had higher three-year mortality than HFpEF	5
Our present study	958 patients into four group: Group 1: low PWV, high LVEF Group 2: low PWV, low LVEF Group 3: high PWV, high LVEF Group 4: high PWV, low LVEF	Median 93 months	Group 4: highest all-cause and CV mortality. Group 1 vs 3: similar overall and CV mortality. Group 2: higher CV mortality than group 3.	

CV: cardiovascular; HFmrEF: heart failure with mid-range ejection fraction; HFpEF: heart failure with preserved ejection fraction; HFrEF: heart failure with reduced ejection fraction; LVEF: left ventricular ejection fraction; MI: myocardial infarction; PWV: pulse wave velocity; Ref: reference.

Ref 1: Sung SH, Yu WC, Cheng HM, et al. Pulsatile hemodynamics and clinical outcomes in acute heart failure. Am J Hypertens. 2011;24(7):775-82.

Ref 2: Demir S, Akpınar O, Akkus O, et al. The prognostic value of arterial stiffness in systolic heart failure. Cardiol J. 2013;20(6):665-71.

Ref 3: Regnault V, Lagrange J, Pizard A, et al. Opposite predictive value of pulse pressure and aortic pulse wave velocity on heart failure with reduced left ventricular ejection fraction: insights from an Eplerenone Post-Acute Myocardial Infarction Heart Failure Efficacy and Survival Study (EPHESUS) substudy. Hypertension. 2014;63(1):105-11.

Ref 4: Tokitsu T, Yamamoto E, Oike F, et al. Clinical significance of brachial-ankle pulse-wave velocity in patients with heart failure with preserved left ventricular ejection fraction. J Hypertens. 2018;36(3):560-568.

Ref 5: Huang WM, Sung SH, Yu WC, et al. Perturbations of pulsatile hemodynamics and clinical outcomes in patients with acute heart failure and reduced, mid-range or preserved ejection fraction. PLoS One. 2019;14(8):e022018.
